# Large Scale Phenotyping Provides Insight into the Diversity of Vegetative and Reproductive Organs in a Wide Collection of Wild and Domesticated Peppers (*Capsicum* spp.)

**DOI:** 10.3390/plants7040103

**Published:** 2018-11-19

**Authors:** Pasquale Tripodi, Barbara Greco

**Affiliations:** Research Centre for Vegetable and Ornamental Crops, CREA, 84098 Pontecagnano Faiano, Italy; barbara.greco@unina.it

**Keywords:** *Capsicum*, wild species, high-throughput phenotyping, plant descriptors, Tomato Analyzer, germplasm diversity

## Abstract

In the past years, the diversity of *Capsicum* has been mainly investigated through genetics and genomics approaches, fewer efforts have been made in the field of plant phenomics. Assessment of crop traits with high-throughput methodologies could enhance the knowledge of the plant phenome, giving at the same time a key contribution to the understanding of the function of many genes. In this study, a wide germplasm collection of 307 accessions retrieved from 48 world regions, and belonging to nine *Capsicum* species was characterized for 54 plant, leaf, flower and fruit traits. Conventional descriptors and semi-automated tools based on image analysis and colour coordinate detection were used. Significant differences were found among accessions, between species and between sweet and spicy cultivated types, revealing a large diversity. The results highlighted how the domestication process and the continued selection have increased the variability of fruit shape and colour. Hierarchical clustering based on conventional and fruit morphological descriptors reflected the separation of species on the basis of their phylogenetic relationships. These observations suggested that the flow between distinct gene pools could have contributed to determine the similarity of the species on the basis of morphological plant and fruit parameters. The approach used represents the first high-throughput phenotyping effort in *Capsicum* spp. aimed at broadening the knowledge of the diversity of domesticated and wild peppers. The data could help to select best the candidates for breeding and provide new insight into the understanding of the genetic base of the fruit shape of pepper.

## 1. Introduction

Pepper (*Capsicum* spp.) is part of the large Solanaceae family, which, among more than 90 genera and 2500 species of flowering plants, includes commercially important vegetables such as tomato, potato and eggplant. The genus has its origins in Central and South American regions and according to recent estimates comprises more than 35 species grouped in 11 clades (or complexes), three of which (Annum, Baccatum and Pubescens) encompass domesticated and wilds relevant in terms of nutritional and economic importance and widely used for genetic improvement [1]. The cultivated pepper (*C. annuum*), which is grown as sweet and hot types in all world regions, and two domesticated species (*C. frutescens* and *C. chinense*) mainly cultivated as spice crops in Africa, Asia and South America, belong to the Annuum complex. *C. baccatum* and *C. pubescens* belong to the homonymous complexes and include types with different levels of spiciness predominantly grown in the Latin American regions [2]. Several other wild relatives within the main clades (*C. annuum* var. *glabriusculum*, *C. chacoense*, *C. eximium*, and *C. praetermissum*) are mainly circumstantiated in the native area of *Capsicum*. After the XVth century, peppers have been spread throughout the tropical and temperate world regions becoming a part of the local cultures and livelihood for many farmers. The domestication and selection processes have allowed the development of an extraordinary variation for plant architecture (e.g., growth habit), vegetative traits (e.g., leaf, stem colour), and fruit features (e.g., shape, size, colour and aroma), which makes peppers suitable for multiple uses. Nowadays, more than 20 market types (bell, cayenne, ancho, jalapeño, pasilla, hungarian wax, jwala, thai, etc.) (Bosland, 1990) are recognized and consumed [3]. This intrinsic variability represents an important resource for breeding and varietal selection in pepper [4].

The diversity in *Capsicum* spp. has been investigated principally at DNA level by means of various type of molecular markers [5,6,7,8,9] including Next Generation Sequencing (NGS) approaches [10]. The evolution of genomics in terms of efficiency of sequencing at affordable costs enhanced the dissection of the molecular diversity with a throughput and precision never reached before. Few efforts have been instead performed for large-scale phenotyping due to the high cost required for automation and technologies. This gap poses the risk of the underutilization of the potentiality stored in genetic resources. Indeed, despite the availability of whole genome sequence in many crops, the lack in precise phenotyping reduces the knowledge of the function of many genes [11]. Depending on the devices and procedures for data analysis and acquisition, phenotyping can be performed with different depth scales and processivity. The morphological characterization is the first step in managing and exploring the features of germplasm resources. In pepper, morphological descriptors for varietal discrimination (Bioversity International) are available and can be used to characterize the vegetative and reproductive part of plants [12]. Although these descriptors are easy to measure, they do not allow the precise assessment of fruit features (size, shape, colour). Moreover, collecting related data is time-consuming and subject to bias. For covering this gap, automated devices can be applied for precise exploration of the phenotypic diversity of crops. In this respect, a freeware for the analysis of the fruit shape (Tomato Analyzer) has been developed in tomato [13,14]. The Tomato Analyzer (TA) performs semiautomatic and high-throughput quantitative measurements of fruit traits from scanned images of fruit sections, eliminating the errors related to subjective scoring. The program allows measuring 38 morphological attributes, determining traits nearly impossible to quantify manually. TA was developed to analyze tomato fruit in order to study the genetic basis of fruit traits and to characterize germplasm collections [13,14,15,16,17,18,19], but it can be used to evaluate the fruits of other species and other plant organs such as seeds, flowers, and leaves [13]. Applications of TA are reported in eggplant to characterize and classify different species and cultivar groups according to fruit shape [20,21].

In *Capsicum*, TA has already been applied to characterize a collection of 116 lines of the cultivated species (*C. annuum*) [22], for QTL mapping in biparental cross [23] and for the identification of molecular candidates of fruit size and shape regulation in 40 lines of *C. annuum* [24]. No applications are reported in large collections of diverse species.

This study investigates a worldwide collection of 307 diverse accessions of nine *Capsicum* species using the Bioversity International descriptors for plant traits and semi automated tools for the assessment of fruit morphology and colour. To achieve this objective, the relationships among species on the basis of phenotypic diversity are determined, and the potentiality of Tomato Analyzer in the wild and domesticated species of pepper is described. The study represents the first high-throughput phenotyping effort for fruit traits in different *Capsicum* species and the gained information contributes to increasing the knowledge of the phenotype, which can be exploited for selection and breeding purposes as well as for future association mapping studies.

## 2. Results

### 2.1. Diversity among Accessions

**Figure 1:**
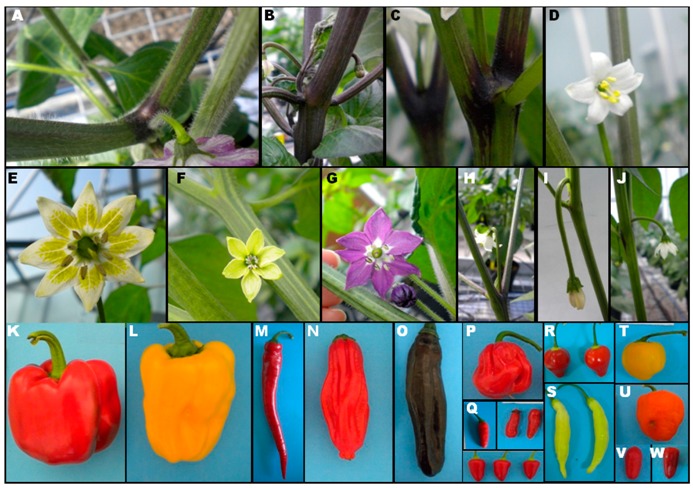
Plant, flower and fruit variability of the pepper collection under study. Stem pubescence in *C. pubescens* (**A**); purple stem colour in *C. chinense* (**B**); nodal anthocyanin in *C. annuum* (**C**); flower of *C. chacoense* with white corolla and yellow anthers (**D**); flower of *C. baccatum* with typical spots (**E**); yellow corolla in *C. frutescens* (**F**); *C. pubescens* flower (**G**); erect flower position in *C. annuum* (**H**); pendant flower position in *C. annuum* (**I**); intermediate flower position in *C. annuum* (**J**); blocky red fruits of sweet *C. annuum* (**K**); blocky yellow fruits of sweet *C. annuum* (**L**); horn-shaped fruit of spicy *C. annuum* (**M**); *C. chinense* fruits (**N**–**P**); *C. frutescens* fruits (**Q**); *C. baccatum* var. *baccatum* fruits (**R**); *C. baccatum* var. *pendulum* fruits (**S**); yellow *C. pubescens* fruits (**T**); orange *C. pubescens* fruits (**U**); *C. chacoense* fruits (**V**); *C. annuum* var. *glabriusculum* fruits (**W**).

The diversity in plant and fruit traits has been firstly explored within genotypes with the aim of identifying traits of interest in each individual. The characterization of the 307 *Capsicum* genotypes revealed a high phenotypic diversity of the collection under study (Figure 1).

**Figure 2:**
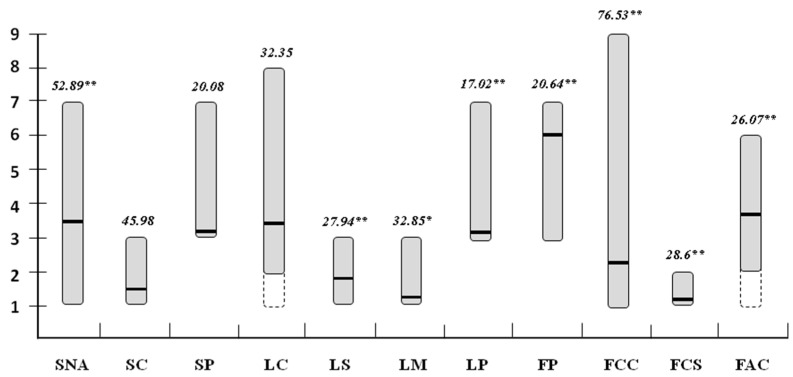
Range (grey bars), means (black lines), coefficient of variation (CV% in italics) and significance (** *p* < 0.01; * *p* < 0.05) of differences between accessions means for 11 Bioversity International traits. The dotted bars indicate the portion of the scale not covered by the accessions studied. SNA = Nodal Anthocyanin, SC = Stem Colour, SP = Stem Pubescence, LC = Leaf Colour, LS = Leaf Shape, LM = Lamina Margin, LP = Leaf Pubescence, FP = Flower Position, FCC = Corolla Colour, FCS = Corolla Shape, FAC = Anther Colour.

Out of the 11 conventional descriptors used, highly significant differences (*p* < 0.01) were found among the means of the individuals for seven traits including Nodal Anthocyanin, Leaf Shape and Leaf Pubescence, and flower descriptors. For Lamina Margin, significant differences at *p* < 0.05 were detected. The remaining traits including the colour and the pubescence of the stem and the colour of the leaves, did not show any significance (Figure 2). For the leaf and anther colour, the range of variation did not cover the entire scale, indeed, the collection did not include accessions with yellow leaves and white anthers. A coefficient of variation up to 76.53% was evidenced for corolla colour while for the remaining traits the CV% was lower than 53%.

Fruit morphological characterization involved the collection of 36 scanned images of fruit sections for each genotype. In total, 11.124 sections were analyzed. As occurred for the plant descriptors, a wide diversity was found for fruit shape parameters in the accessions studied. Highly significant differences (*p* < 0.0001) were evidenced for the 38 Tomato Analyzer descriptors. All fruit size traits, as well as curved Fruit Shape Index and Circular and Lobedness Degree, were those explaining the greater part of variation as shown by the F values (Table 1). Five traits, including Perimeter, Width mid-height, Maximum height, Curved height, and Circular, evidenced an R square greater than 0.90. For each category, based on the coefficient of variation, the descriptors with largest variability were: H. Asymmetry Ob (556.95%), Distal Indentation Area (321.27%), Proximal Indentation Area (201.39%), Distal eccentricity (184.06%), Area (98.98%), Lobedness Degree (76.81%), Curved Fruit Shape Index (75.49%), Fruit Shape Triangle (66.58%), Circular (48.15%). A minimum value of zero was observed in three asymmetry descriptors (Obovoid, Ovoid, and H. Asymmetry Ob) and for Distal Indentation Area.

For the analysis of CIELab coordinates over 1000 fruits were measured. All colour traits were highly significant in the collection studied. A larger amount of the redness and yellowness components were evidenced as shown by the a* and b* ranges, respectively. L* and chroma values covered about the 80% and 96% of the respective range, evidencing a higher amount of lightness and intense saturation of colours. Overall, the collection ranged from green to violet as shown by the Hue Angle values range.

### 2.2. Diversity between Capsicum Species

Diversity within species was assessed in order to give insight into their relationship and determine traits to be exploited in each gene pool. The results of a one-way ANOVA are reported in Table S1. All leaf and flower traits were significantly different between species, with the exception of leaf colour. Stem traits did not show any significance, with the exception of Nodal Anthocyanin. Accessions of *C. annuum*, *C. baccatum* var. *pendulum*, and *C. chinense* evidenced variation in flower position; furthermore, the first two species showed a high variation for corolla colour. The post hoc Tukey test showed significant differences for conventional descriptors among the nine species.

Nodal anthocyanin differentiated *C. annuum* and *C. chinense* from the rest. Leaf pubescence and corolla colour revealed differences between *C. pubescens* and the remaining species (Figure 1).

For fruit features, a wide variability was found in domesticated species, while lower average values were evidenced in the wilds. Highly significant differences (*p* < 0.001) were found between the average values for 34 out of the 38 Tomato Analyzer descriptors (Table S1). No significant differences were found for Proximal and Distal Eccentricity, while significant differences at *p* < 0.01 and *p* < 0.05 were found for H. Asymmetry Ob and Proximal Angle Micro, respectively. For all fruit size traits, *C. annuum* statistically diverged from the other species, exhibiting the highest maximum values. Among domesticated species, *C. frutescens* showed the smallest mean values for fruit size traits (Perimeter, Area, Width, and Height) while *C. baccatum* var. *pendulum* evidenced the highest mean values of the fruit shape external indices (2.88 and 3.43 for FSEI and FSEII, respectively). Several traits did not show any significativity in wild species: V. Asymmetry and Pericarp Thickness in *C. annuum* var. *glabriusculum*; Obovoid, H. Asymmetry.ob and Width Widest Pos in *C. chacoense*. In both species, all Proximal and three Distal fruit end-shape as well as Proximal and Distal eccentricity were not significant. The number of significant differences was variable in the domesticated species, being 37 in both *C. baccatum* var. *pendulum* and *C. frutescens*, 36 in *C. pubescens* and 35 in *C. baccatum* var. *baccatum*. Only for *C. annuum* and *C. chinense* all traits were statistically significant, although in the latter species, some evidenced a *p* less than 0.05. A normal distribution was shown by Eccentricity Area Index. Several morphological traits, including fruit size, shape, and blockiness, showed a positive skewed distribution (Figure S1). For various proximal and distal fruit end shape, a bimodal or trimodal distribution was observed. A negative skewed distribution was exhibited by Pericarp Thickness and Eccentricity.

CIElab coordinates (Table S1) revealed a divergence for L* space of about 10% among accessions with low values (darker fruits) in *C. annuum* and *C. chacoense*, and high values (brightest colours) in *C. pubescens* and *C. baccatum* var. *pendulum*. The wild species presented more intense red colour exhibiting average a* values higher than domesticated ones. More vivid external fruit colour was observed in *C. baccatum* var. *pendulum* and *C. eximium*, as shown by chroma values (Table S1). All species were included in the orange hue angle range. The highest and the lowest proportions of red were evidenced by *C. chacoense* and *C. pubescens*, respectively. A normal distribution was evidenced for chroma while in the other colour coordinates a skew was observed.

**Figure 3:**
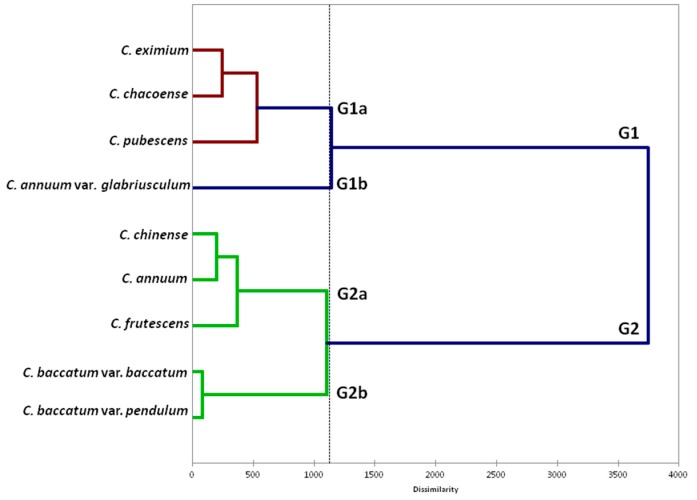
Cluster analysis (Ward coefficient) based on plant and fruit traits for the nine *Capsicum* species evaluated in the present study.

Hierarchical clustering based on conventional descriptors and fruit morphological traits separated the species into two main groups (G). The former (G1) included the wild species and *C. pubescens*, the latter (G2), the remaining cultivated and domesticated ones (Figure 3). In the first group, *C. annuum* var. *glabriusculum* clustered separately from the other species. The second main group (G2) was subdivided into two subclusters: G2a, including *C. annuum*, *C. chinense* and *C. frutescens*, and G2b including the two forms of *C. baccatum*

### 2.3. Diversity between Sweet and Hot Cultivated Pepper Types

Differences between hot and sweet types have been assessed in order to determine major differences among the most commercially consumed peppers. Significant differences (*p* < 0.05) between sweet and hot peppers were found only for two plant traits, including leaf colour and corolla colour (Table 2). All the sweet pepper genotypes under study presented green leaves and corolla colour ranging from white to light yellow. Moreover, no pubescence in the stem and in the leaves, as well as spot colour within the corolla, was observed. In hot types, the variation in most of the traits covered the whole scale, except leaf colour, which ranged from light green to variegated. No ciliated lamina margin was observed in the leaves of both sweet and hot accessions. For fruit traits, highly significant differences (*p* < 0.001) between sweet and hot types were found for 32 descriptors and significant differences at P less than 0.05 were found for Distal Fruit Blockiness and Obovoid (Table 2). Hot accessions presented smaller fruits, larger fruit shape indices, higher values for Blockiness, and more Internal Eccentricity. Proximal and Distal Fruit End Shapes were instead greater in sweet accessions with the exception of Distal End Protusion. For most of the traits, a higher coefficient of variation was found within the hot genotypes. Significant differences were found for L*, a*, and chroma, and a greater variation for the CIELAB coordinates was evidenced in sweet accessions. Hot types presented a more intense red colour than sweet types, as evidenced by the lower L* value and the greater a* and chroma values.

### 2.4. Multivariate Analyses

**Figure 4:**
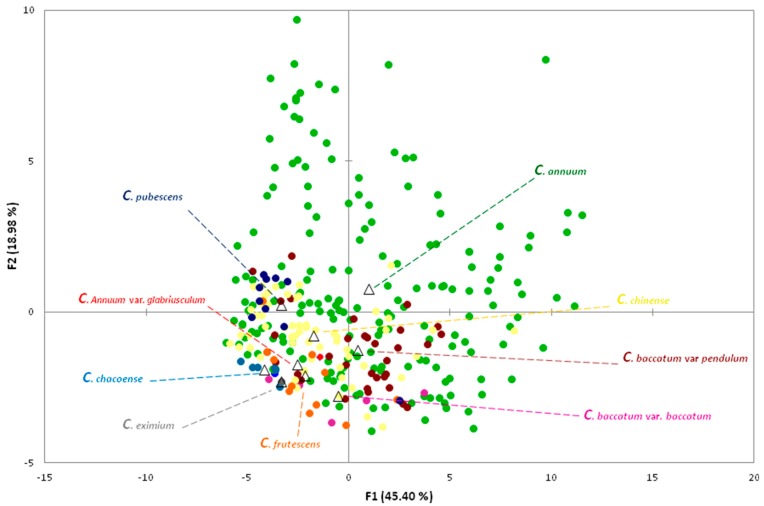
Loading plot of the first and second principal component of 38 Tomato Analyzer descriptors surveyed in the 307 genotypes studied belonging to 9 pepper species. Accessions of each species are represented by dots of different colours. First and second component centroids for each Capsicum species are indicated by a triangle.

**Figure 5:**
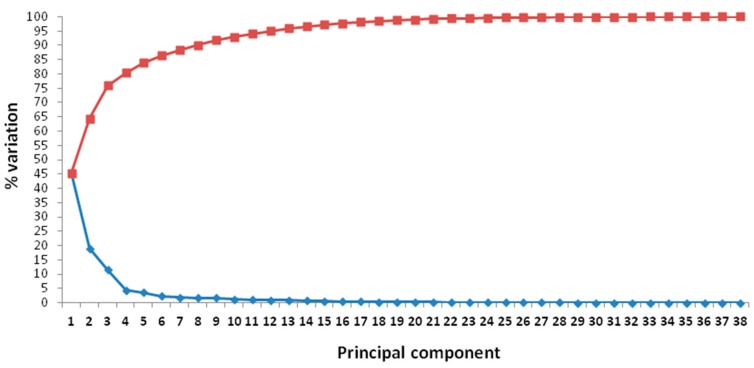
Principal components and related percentage of variation explained. The red line (above) indicates the cumulative variation of the components from 1 to 38. The blue line (below) indicates the variation explained by each component.

Multivariate analysis has been performed on fruit morphological traits given the enormous variability found in the studied accessions. The PCA in the first two dimensions explained 64.38% of the total variance among accession means (Figure 4; Table S2) while the remaining 35.62% of the variation was explained by the other components (Figure 5; Table S3). Overall, 90% of variation was explained by the first eight components (Figure 5).

The first component accounting for 41.40% of the total variance was positively correlated with Fruit Size, Shape, Blockiness, Homogeneneity, Internal Eccentricity, and Latitudinal Section traits with the exception of Width Mid-Height, Rectangular, Eccentricity, and Pericarp Thickness, and negatively correlated with Proximal and Distal Fruit End Shape, with the exception of Shoulder Height and Distal End Protusion (Table S2). Within the Asymmetry traits, only Ovoid, V. Asymmetry, and H. Asymmetry Ov were positively correlated to the first component. The second component which explained 18.98% of the total variance was positively correlated with all Fruit Size and Proximal Fruit End Shape traits, and negatively correlated with all Fruit Shape Index traits. Overall, 14 out of the 38 Tomato Analyzer descriptors resulted in being positively correlated to the first and second component, while only two (Pericarp Thickness and Eccentricity) were negatively correlated to both components (Table S2). Three fruit shape index traits (External II, Curved, and Internal), Circular, H. Asymmetry Ov, and Lobedness Degree were the main factors discriminating the genotypes under study on the first axis, each accounting for over 5% of total variation and showed a correlation higher than 0.9 (Table S2). On the second axis, two fruit size traits (Maximum Width, Width-Mid Height) and Pericarp Area were the main factors discriminating the accessions, and each accounted for over the 11% of the total variation, although only the first two exhibited a very high correlation (>0.9). The projection of the accessions on the two-dimensional PCA graph confirmed the wide variability for fruit-related traits, particularly for the cultivated species which had a high dispersion. Indeed, the accessions of *C. annuum* plotted in different areas of the chart. Accessions of the wild species, having the smallest fruits, plotted in the negative area of both first and second components while all *C. frutescens* genotypes and most of the *C. chinense* accessions were all concentrated on the negative axis of the first component. Most of the *C. baccatum* accessions presented positive values for the first component and negative values for the second one. Contrariwise, *C. pubescens* genotypes were characterized by negative values for the first component and positive values for the second one.

**Figure 6:**
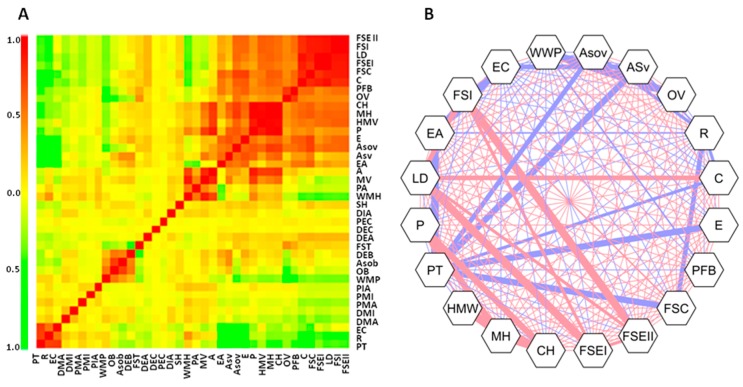
(**A**) Heatmap of correlation for 38 Tomato Analyzer descriptors. Positive correlations are displayed in red and negative correlations in green. Colour intensity is proportional to the correlation coefficients. On the left side of the correlogram, the legend colour shows the correlation coefficients and the corresponding colours. (**B**) Correlation network for most significant traits (*p* < 0.05) using Pearson test after Bonferroni correction. Positive correlations are displayed in red and negative correlations in blue colour. Thicker lines indicate significant correlations. The strength of the correlation is indicated by the thickness of the lines.

The network of correlation for fruit morphological traits revealed how some were rather independent, whereas a group of traits clustered together because of a reciprocal tight correlation (Figure 6). Strong positive significant correlations were evidenced between fruit shape (FSI, FSEI, FSEII) and size traits (P, MH, CH, HMW), indicating, as expected, that larger fruits had larger dimensions of width and height axes. Lobedness Degree showed positive correlation with Fruit shape external I and Circular. Negative significant correlations were evidenced within Asymmetry traits (WWP, ASov, ASv, OV) and between Curved Fruit Shape Index and Rectangular. Pericarp Thickness was negatively correlated with Asymmetry traits (ASov and ASv), Homogeneity traits (C and E), Eccentricity and Curved Fruit Shape, indicating that the pericarp was thicker in not rectangular fruits.

In order to identify the most important traits which determine the shape of fruit and are able to discriminate the species, we further selected: (i) eight fruit morphological traits having a high correlation to the first two principal components and mainly contributing to the variance explained (Width-Mid Height, Maximum Width, Fruit Shape Index External II, Curved Fruit Shape Index, Circular, H. Asymmetry Ov, Fruit Shape Index Internal, and Lobedness Degree, Table S2); (ii) two plant descriptors exhibiting the greatest variation between species (Nodal Anthocyanin and Corolla Colour) (Figure 2 and Table S1). PCA was inferred by means of these traits, confirming how this minimal set contributes to the maximum of the variation on the first two components (Figure S2a). On the basis of the PCA it was not possible to accurately discriminate the species except for the *C. chacoense* (Figure S2b). Hierarchical clustering allowed instead a better distinction of the wild species from the rest (Figure S3).

## 3. Discussion

Understanding and utilizing crop diversity through extensive phenotyping is pivotal for breeding, conservation, and management of genetic resources. In pepper, fruit morphology and colour are the main attributes to be considered for market types definition, thus being major objectives to pursue for varietal selection. In the present study, a very large collection of nine *Capsicum* species, was investigated for 54 plant and fruit traits by means of common descriptors and semi automatic high-throughput techniques, allowing collection of over 450,000 phenotypic data points. A lack of large-scale phenotyping studies occurs for pepper and this research aim to cover the gap, representing the first attempt to deeply assess a broad collection in terms of numbers of accessions and diversity enclosed. The phenotypic variation of the collection was firstly investigated between species. Although a no balance in terms of numbers of accessions for species must be recognized, it must be taken into account the difficulty in retrieving sources of germplasm in particular for wild relatives. Moreover, considering that most of the variation for fruit morphology occurs in *C. annuum* (cultivated type), an in-depth analysis between sweet and spicy types (assessed by tasting ripe fruits according to Bioversity International) was subsequently performed within this species. All traits exhibited a wide diversity among genotypes. Considering the existence of specific conventional descriptors distinguishing different pepper species (i.e., black seeds in *C. pubescens* or green corolla spot in *C. baccatum*), attentiveness was focalized on those most interesting for breeding purposes such as colours and pubescence of stem and leaves. Anthocyanins in leaves can significantly influence the response to biotic and abiotic stresses, being involved in defence mechanisms against various pathogens and exhibiting tolerance to many kinds of environmental stressors [25]. Certain insect, for example avoid eating red-pigmented leaves which can result in being inedible. Moreover, anthocyanic cell vacuoles, by intercepting the high-energy quanta, can prevent photolysis of light-sensitive chemicals in plants [26]. Moreover, anthocyanins are pigments responsible for fruit colour and display important nutraceutical properties and antioxidant capacity [4]. The pubescence of stem and leaves due to the presence of glandular trichomes has been demonstrated to play a role in defence against insect and herbivores and various evidences are reported in Solanaceae [27]. Interestingly, in this study, accessions with high pigment content as well dense pubescence in the vegetative parts were found within the cultivated species. The possibility of transferring these traits within the same gene pool, avoiding interspecific crosses, reduces the occurrence of sterile hybrids and segregation distortion, and avoids the use of aids such as embryo rescue [28,29]. Most of the differences among species were observed for fruit traits. The imaging tool clearly distinguished, as expected, the wild species from domesticated ones based on fruit size and fruit shape traits. As evidenced by the two-dimensional PCA, the cultivated species had the highest values and the biggest variability for these descriptors, confirming that the domestication and the continued selection have resulted in a large increase of shape variation of pepper fruits [30]. The TA was useful to compare wild types, since despite the small fruits, it was possible to observe a more triangular shape for *C. annuum* var. *glabriusculum* and more elongated-heart shape for *C. chacoense*. Indeed, the former was distinguished for Fruit Shape Triangle, the latter for Obovoid and Width Widest Pos. Most of the variation between hot and sweet types was due to fruit morphology, showing the sweet types with greater sizes and more regular shapes, while spicy types evidenced smaller fruits with triangular and circular shapes. In this study, the performed TA assessment showed various traits not significant or with a low significance level, such as Proximal and Distal Eccentricity, Proximal Angle Micro, H. Asymmetry Ob, according to previous studies performed in diverse collections of eggplant [20,31] and tomato [19]. Moreover, four traits, including Area, Fruit Shape Triangle, Circular, and Proximal Indentation Area, were those with the largest variation for each of the descriptors categories in agreement with evidences in tomato [19]. In addition, the variation among species was not considerable if considering some descriptors (i.e., Proximal and Distal Eccentricity, Shoulder Height, Proximal Angle Micro, Distal Indentation Area, H. Asymmetry Ob, Pericarp Thickness), indicating that TA is not able to perform a precise characterization on these traits and also suggesting a low selection pressure on these.

The variability of pepper fruit colour, due to various mutations [32], is related to the accumulation of different bioactive compounds, which make *Capsicum* a good source of antioxidants as well as suitable for different uses. The domestication process resulted in the development of various ranges of colours in all domesticated and cultivated species [30]. As observed in our study, the wild species evidenced a low variability in all colour components, evidencing a higher redness than the rest of the collection. On the contrary, a wide colour range was observed in *C. annuum* in both sweet and hot types, although the latter evidenced a major redness and colour saturation suggesting a higher amount of red carotenoid pigments such as capsanthin and capsorubin [33].

The dendrogram derived from the combining of conventional and TA descriptors reflected the separation of the domesticated and cultivated species according to the existing complexes. Indeed, species within the Annuum complex (*C. annuum*, *C. frutescens*, and *C. chinense*) were grouped together and separately from the rest. *C. baccatum* and *C. pubescens*, which represent diverse taxons from the Annuum complex, formed separate clusters, although the latter was unexpectedly clustered with wild species (Figure 3). Hierarchical analysis reflected also the crossability between gene pools linked to the unilateral incompatibility between the *C. pubescens* and all other species of *Capsicum* and the possibility of overcoming the incompatibility barriers between the Annuum and the Baccatum complexes through the use of bridge species or embryo rescue [2,29]. Considering that the aim of this study is not to give insight into the phylogenesys and/or domestication of *Capsicum*, we could suggest that the flow among distinct gene pools could have contributed determinin the similarity of the species based on morphological and fruit shape parameters. However, further detailed analysis needs to be performed in order to confirm this hypothesis, considering other factors such as the parallel variation in response to human and/or natural selection.

From PCA analysis, the identification of a minimal set of 10 plant and fruit traits allowed a better discrimination of wild species, being unable instead to clearly distinguish the domesticated and cultivated ones. This could be explained by the complex variability within the latter. Nevertheless, index traits were those mostly variable between accessions and species and can be considered the most relevant for precision breeding. Further experiments which include selected accessions and their hybrids could help to develop a model for fruit shape prediction.

Beyond the scope of characterization, the approach used could give more insight into the understanding of the genetic base of fruit shape in pepper. To date, various researches have been performed involving various bi-parental intra- and interspecific mapping populations [23,34,35,36,37,38] reporting the existence of different QTLs with minor or large effect. These mapping populations, although informative, have the disadvantage of capturing only the variation of the two parents and can be affected by lack of recombination occurring in the interspecific hybridization. The possibility to implement high-throughput genotyping and phenotyping in genome-wide association studies to investigate the existing variation in large collections could give novel insight into the understanding of the genetic basis of traits involved in the fruit morphology of pepper. Moreover, morphological traits can corroborate genetic data in the assemblage of the core collections and provide information of parental performance to be used in a breeding program [39].

Toward this objective, the phenomic analysis carried out in the present study could be integrated with genomic analysis of the characterized collection. The observed correlations between pairs of morphological traits suggest that according to the TA, category, size, shape, homogeneity, and asymmetry of fruits are traits to focus on in order to obtain desired shapes. These evidences are highly interesting if considering the market destinations of peppers and the expansion of packaged products, indicating TA as a useful tool to predict fruit shape in breeding programs, due to the precise morphological characterization performed of fruits in pepper.

## 4. Materials and Methods

### 4.1. Plant Material

A collection of 307 diverse accessions sampled from 48 world countries (Table 3) and belonging to cultivated and domesticated species (*C. annuum* var. *annuum*, n° 180, *C. baccatum* var. *baccatum* n° 5, *C. baccatum* var. *pendulum* n° 33, *C. chinense* n° 57, *C. frutescens* n° 12, *C. pubescens*, n° 10) as well wild species (*C. annuum* var. *glabriusculum*, n° 2, *C. chacoense* n° 7, *C. eximium* n° 1) were used in the present study. Genotypes were selected avoiding any duplications from two main European germplasm banks (The Centre for Genetic Resources, CGN, Wageningen, The Netherlands, and the Leibniz-Institut für Pflanzengenetik und Kulturpflanzenforschung, IPK, Gatersleben, Germany), seed companies, local farmers, and associations, and were priorly subjected to two cycles of controlled self-fertilization. For each accession, three plants were grown in controlled environmental conditions in the greenhouse of the Research Centre for Vegetable and Ornamental Crops following a completely randomized design.

### 4.2. Phenotypic Characterization

For the plant traits characterization, eleven qualitative/pseudo-qualitative descriptors were assessed in individual plants for each accession following the protocol proposed for *Capsicum* by the IPGRI/Biodiversity (1995) [11]. Three category of traits were analyzed: (i) stem traits, including stem Nodal Anthocyanin (SNA) (0; 1, green; 3, light purple; 5, purple; 7, dark purple), Stem Colour (SC) (1, green; 2, green with purple stripes; 3, purple), Stem Pubescence (SP) (3, sparse; 5, intermediate; 7, dense); (ii) leaf traits, including Leaf Colour (LC) (1, yellow; 2, light green; 3, green; 4, dark green; 5, light purple; 6, purple; 7, variegated; 8, other), Leaf Shape (LS) (1, deltoid; 2, ovate; 3, lanceolate), Lamina Margin (LM) (1, entire; 2, undulate; 3, ciliate), Leaf Pubescence (LP) (3, sparse; 5, intermediate; 7, dense); (iii) flower traits, including Flower Position (FP) (3, pendant; 5, intermediate; 7, erect), Corolla Colour (FCC) (1, white; 2, light yellow; 3, yellow; 4, yellow-green; 5, purple with white base; 6, white with purple base; 7, white with purple margin; 8, purple; 9, other), Corolla Shape (FCS) (1, rotate; 2, campanulate), Anther Colour (FAC) (1, white; 2, yellow; 3, pale blue; 4, blue; 5, purple).

**Figure 7:**
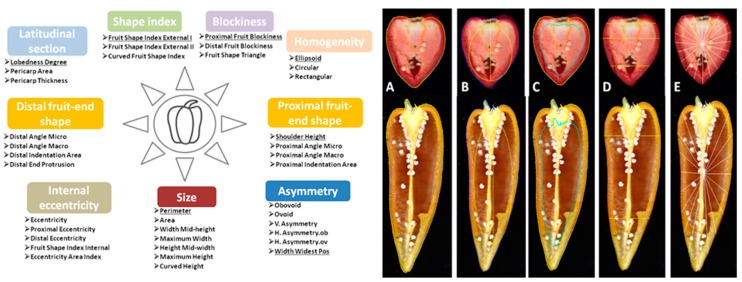
On the right: category of traits scored using Tomato Analyzer (more details in Brewer et al., 2009). On the left: examples of graphical representation of fruit morphology attributes. (**A**) Perimeter, (**B**) internal eccentricity, (**C**) pericarp area, (**D**) obovoid and widest width position, (**E**) lobedness degree.

At maturity stage, six fruits for each plant (18 for each accession) were harvested and subjected to automated phenotyping. The fruits sections obtained after a longitudinal cut were scanned with a CanoScan LiDE 210 photo scanner (Canon, Tokyo, Japan) at a resolution of 300 dpi. Morphometric analysis was carried out using the Tomato Analyzer v 3.0 software (Brewer et al. 2006). Thirty-eight quantitative descriptors, categorized into: fruit size (7), shape index (3), blockiness (3), homogeneity (3), proximal fruit end-shape (4), distal fruit end shape (4), asymmetry (6), internal eccentricity (5), latitudinal section (3), were automatically recorded. Points were adjusted manually when the software was unable to accurately identify the outline of a trait. The categories of the traits assessed are in Figure 7. Details of descriptors are available at the software page [40].

CIELAB (L*a*b*) fruit colour coordinates were measured using a CR-210 Chroma Meter (Minolta Corp., Osaka, Japan) on a sample of five fruits. Measurement was done at the midpoint between the distal and the basal ends of the fruit and expressed as L*, a*, b* values. L* indicates lightness/darkness (0 = black, 100 = white), a* describes intensity in green−red (where a positive number indicates redness and a negative number indicates greenness), and b* describes the intensity in blue−yellow (where a positive number indicates yellowness and a negative number indicates blueness). Chroma (C) [(a*)2 + (b*)2]0.5 and hue angle (h) (h = arctan b*/a*) were estimated by the a* and b* values. Chroma indicates colour saturation. Hue is a measure of an angle (0° or 360° indicates red hue, while angles below 270°, 180°, and 90° indicate blue, green, and yellow hue, respectively).

### 4.3. Data Analyses

All phenotypic traits were subjected to analysis of variance (ANOVA) test. Mean and range values were calculated for each accession and species. Significant differences among species means were detected using Tukey HSD (honest significant difference) test. Results with *p* < 0.05 were considered statistically significant. Coefficient of variation (CV) in percentage was expressed as the ratio of the standard deviation to the mean value multiplied by 100. Experimental data were statistically elaborated by using the statistical software package JMP v7.0 software package (SAS Institute, Cary, NC, USA). Similarity among species based on plant and fruit traits was estimated by agglomerative hierarchical cluster analysis (HCA) using the Ward’s coefficient. Correlations across the genotypes for fruit traits were calculated using Pearson’s test at P less than 0.05 after Bonferroni’s correction for multiple comparisons [41]. The correlogram and the graphical presentation of the network were constructed with the Cytoscape 3.5.1 plug-in Metscape [42,43]. Principal component analysis (PCA) was carried out to determine which are the most effective fruit descriptors in discriminating among accessions using the computer package XLSTAT 2012.1.

## 5. Conclusions

The present study aimed to investigate the plant and fruit characteristics of a wide collection of the main *Capsicum* species. Besides the information on the phenotype, the relationships between the pepper complexes have been investigated evidencing similarities within the species of the same complex based on the morphological traits and confirming how domestication and selection have contributed to broadening the variability particularly for fruit characteristics. The information gained from the present investigation represents the frame for a precise dissection of the genetic basis of fruit traits, which are the main target to pursue in pepper breeding.

## Figures and Tables

**Table 1:** Analysis of variance, mean, range, and coefficient of variation for Tomato Analyzer descriptors and CIELAB coordinates in 307 *Capsicum* accessions.

Traits	Acronim	MS ^a^	TSS%	R	F Value ^b^	Mean	Range	CV
Fruit descriptors								
Perimeter	[P]	3880.00	3.44	0.94	467.85 *	18.99	67.45–2.06	60.53
Area	[A]	6222.19	5.52	0.89	248.87 *	15.10	133.77–0.26	98.98
Width Mid-height	[WMH]	88.61	0.08	0.94	462.31 *	2.51	15.91–0.41	69.24
Maximum Width	[MW]	102.74	0.09	0.89	236.49 *	3.15	18.33–0.49	61.13
Height Mid-width	[HMW]	373.94	0.33	0.85	169.69 *	5.92	22.9–0.42	63.48
Maximum Height	[MH]	532.37	0.47	0.93	417.51 *	6.72	26.34–0.68	63.58
Curved Height	[CH]	566.22	0.50	0.94	457.78 *	7.11	26.88–0.8	61.82
Fruit Shape Index External I	[FSEI]	37.01	0.03	0.76	95.73 *	2.34	12.52–0.37	53.31
Fruit Shape Index External II	[FSEII]	104.25	0.09	0.74	87.64 *	2.91	25.76–0.2	72.8
Curved Fruit Shape Index	[FSC]	194.28	0.17	0.87	210.26 *	3.53	25.07–0.42	75.49
Proximal Fruit Blockiness	[PFB]	1.17	0.00	0.55	36.78 *	0.90	3.23–0.03	28.92
Distal Fruit Blockiness	[DFB]	0.42	0.00	0.27	11.16 *	0.58	5.04–0.02	38.77
Fruit Shape Triangle	[FST]	7.54	0.01	0.17	6.39 *	1.77	56.5–0.04	66.58
Ellipsoid	[E]	0.04	0.00	0.64	54.85 *	0.09	0.27–0.01	47.2
Circular	[C]	0.47	0.00	0.92	345.14 *	0.26	0.5–0.02	48.15
Rectangular	[R]	0.19	0.00	0.51	31.83 *	0.41	0.72–0.02	27.00
Shoulder Height	[SH]	0.01	0.00	0.16	5.74 *	0.02	0.24–0	136.35
Proximal Angle Micro	[PMI]	16,146.46	14.33	0.07	2.15 *	127.01	359.2–0	69.42
Proximal Angle Macro	[PMA]	25,968.55	23.05	0.18	6.45 *	102.64	353.8–0	67.01
Proximal Indentation Area	[PIA]	0.01	0.00	0.15	5.29 *	0.02	0.67–0	201.39
Distal Angle Micro	[DMI]	18,696.80	16.60	0.11	3.72 *	107.43	359.7–0	68.78
Distal Angle Macro	[DMA]	28,587.58	25.38	0.23	9.05 *	83.39	357.2–0	75.58
Distal Indentation Area	[DIA]	0.01	0.00	0.19	7.30 *	0.01	1.51–0	321.27
Distal End Protrusion	[DEA]	0.32	0.00	0.15	5.47 *	0.13	9.62–0	193.1
Obovoid	[OB]	0.05	0.00	0.21	8.02 *	0.02	1.01–0	360.61
Ovoid	[OV]	0.51	0.00	0.53	34.21 *	0.30	0.89–0	58.86
V. Asymmetry	[ASv]	2.24	0.00	0.58	41.23 *	0.29	6.41–0	123.48
H. Asymmetry.ob	[ASob]	0.55	0.00	0.17	6.04 *	0.06	6.15–0	556.95
H. Asymmetry.ov	[Asov]	17.47	0.02	0.66	58.39 *	0.77	7.83–0	119.57
Width Widest Pos	[WWP]	0.30	0.00	0.35	16.31 *	0.35	0.99–0.02	47.22
Eccentricity	[EC]	0.11	0.00	0.32	14.39 *	0.72	0.8–0.02	14.25
Proximal Eccentricity	[PEC]	0.18	0.00	0.05	1.75 *	0.90	24.67–0.02	36.27
Distal Eccentricity	[DEC]	5.95	0.01	0.07	2.09 *	0.93	166–0.14	184.06
Fruit Shape Index Internal	[FSI]	106.52	0.09	0.73	80.38 *	2.94	26.35–0.2	73.65
Eccentricity Area Index	[EA]	0.15	0.00	0.46	25.33 *	0.45	0.99–0	22.47
Lobedness Degree	[LD]	11,008.87	9.77	0.81	129.89 *	27.12	189.84–1.07	76.81
Pericarp Area	[PA]	0.03	0.00	0.42	21.86 *	0.57	1.22–0.54	8.06
Pericarp Thickness	[PT]	0.01	0.00	0.51	31.22 *	0.22	0.26–0.05	11.51
**Color descriptors**								
L *	[L]	1673.04	9.47	0.87	100.39 *	42.50	97.65–13.31	26.18
a *	[a]	1733.39	9.81	0.85	82.84 *	27.65	58.33–(−19.61)	41.50
b *	[b]	3851.92	21.80	0.93	185.38 *	27.15	95.72–(−9.48)	60.36
Chroma	[CHR]	2746.17	15.54	0.88	102.13 *	41.22	96.88–0.62	34.55
Hue angle	[HA]	7666.55	43.38	0.75	42.55 *	35.89	89.90–(−89.98)	71.80

^a^*MS* mean squares, *TSS* total sum of squares, *R* Rsquare, *F* Fvalue; ^b^ * indicate significant at *p* < 0.0001.

**Table 2:** Mean, range, coefficient of variation (CV%), and significance of differences between means of sweet and hot cultivated peppers for plant traits (IPGRI), fruit descriptors (Tomato Analyzer) and colour traits (Minolta colorimeter).

Plant Descriptors	*C. Annuum* Sweet (*n* = 45)			*C. Annuum* Hot (*n* = 135)			Prob > F
	Mean	Range	CV	Mean	Range	CV	
[SNA]	3.86	7–1	42.25	4.17	7–1	37.40	0.336
[SC]	1.42	3–1	40.60	1.49	3–1	43.93	0.635
[SP]	3.00	3–3	0.00	3.21	7–3	29.90	0.113
[LC]	3.00	3–3	0.00	3.50	8–2	37.73	0.042
[LS]	1.90	3–1	17.40	2.00	3–1	24.60	0.732
[LM]	1.10	2–1	29.00	1.10	2–1	29.00	0.969
[LP]	3.00	3–3	0.00	3.10	7–3	21.60	0.259
[FP]	6.00	7–3	20.30	5.80	7–3	24.60	0.323
[FCC]	1.30	2–1	35.60	2.10	9–1	99.00	0.022
[FCS]	1.01	2–1	16.70	1.02	2–1	11.20	0.553
[FAC]	3.91	5–2	14.36	4.01	5–2	13.70	0.384
**Fruit descriptors**							
[P]	33.58	66.58–6.39	26.72	20.24	67.45–3.57	58.89	<0.001
[A]	42.27	133.77–0.36	41.36	14.27	72.54–0.36	86.37	<0.001
[WMH]	6.06	15.91–0.62	36.23	2.06	7.7–0.46	49.23	<0.001
[MW]	6.85	18.33–0.71	29.95	2.91	13.17–0.71	49.34	<0.001
[HMW]	8.79	21.32–0.67	39.83	6.55	22.9–0.42	64.41	<0.001
[MH]	9.90	22.19–1.02	36.72	7.63	26.34–0.94	63.11	<0.001
[CH]	10.89	22.59–2.09	33.56	7.96	26.88–1.2	61.47	<0.001
[FSEI]	1.60	5.05–0.37	50.43	2.73	12.52–0.37	51.32	<0.001
[FSEII]	1.80	12.99–0.29	74.17	3.65	25.76–0.2	70.58	<0.001
[FSC]	2.22	15.97–0.42	74.45	4.54	25.07–0.42	71.79	<0.001
[PFB]	0.88	2.3–0.08	30.83	0.99	2.76–0.03	27.48	<0.001
[DFB]	0.61	3.21–0.08	37.43	0.60	5.04–0.04	41.91	0.018
[FST]	1.58	11.79–0.05	48.76	1.85	20.89–0.05	56.57	<0.001
[E]	0.09	0.23–0.01	40.85	0.10	0.27–0.01	47.91	<0.001
[C]	0.20	0.48–0.02	52.01	0.30	0.5–0.02	42.58	<0.001
[R]	0.48	0.72–0.05	20.05	0.39	0.7–0.02	30.70	<0.001
[SH]	0.03	0.24–0	109.88	0.03	0.19–0	132.82	<0.001
[PMI]	145.56	357.8–0	69.86	122.20	359.2–0	72.94	<0.001
[PMA]	133.53	352.1–0	67.57	96.83	353.8–0	71.95	<0.001
[PIA]	0.04	0.67–0	190.88	0.02	0.35–0	160.20	<0.001
[DMI]	127.60	356.7–0.2	64.50	104.79	359.7–0	68.31	<0.001
[DMA]	99.24	351.7–0	67.05	79.03	357.2–0	84.98	<0.001
[DIA]	0.02	0.66–0	264.11	0.01	1.51–0	401.60	<0.001
[DEA]	0.09	1.15–0	216.62	0.14	9.62–0	218.36	<0.001
[OB]	0.03	0.71–0	306.91	0.02	1.01–0	484.94	0.015
[OV]	0.27	0.82–0	64.86	0.36	0.89–0	48.62	<0.001
[ASv]	0.42	6.41–0.02	118.12	0.34	4.53–0.01	119.48	<0.001
[ASob]	0.07	5.38–0	509.16	0.05	6.15–0	716.74	0.15
[Asov]	0.81	6.91–0	101.32	1.05	7.83–0	108.57	<0.001
[WWP]	0.35	0.9–0.04	44.15	0.30	0.99–0.02	55.78	<0.001
[EC]	0.71	0.8–0.12	12.16	0.70	0.8–0.02	18.51	0.29
[PEC]	0.89	3.61–0.14	12.37	0.90	12.31–0.02	35.58	0.28
[DEC]	0.90	5.51–0.5	17.68	0.98	166–0.18	263.60	0.33
[FSI]	1.81	13.15–0.29	74.36	3.68	26.35–0.2	71.37	<0.001
[EA]	0.40	0.95–0	29.05	0.48	0.99–0	23.09	<0.001
[LD]	15.90	74.81–1.07	78.41	34.20	189.84–1.07	68.83	<0.001
[PA]	0.63	1.22–0.56	16.24	0.56	1–0.54	3.95	<0.001
[PT]	0.23	0.25–0.06	9.08	0.22	0.26–0.05	14.01	<0.001
**Colour descriptors**							
L *	41.28	92.65–23.58	26.20	38.65	78.46–13.31	19.62	<0.001
a *	19.29	45.69–(−19.61)	69.84	30.26	54.54–(−18.45)	23.75	<0.001
b *	22.99	82.33–2.26	66.38	22.31	83.62–(−9.48)	51.54	0.284
Chroma	33.86	82.34–2.55	38.28	38.67	83.62–8.94	26.10	<0.001
Hue angle	32.11	89.77–(−89.07)	107.50	32.44	89.79–(−89.63)	48.77	0.756

**Table 3:** Capsicum species and provenance of the accessions considered in the present study. The number of the accessions for each country is in brackets.

Species.	Country Regions *
*C. annuum*	Brazil (1), Bulgaria (1), France (1), India (1), Japan (1), Kosovo (1), Mauritius Island (1), Nepal (1), Pakistan (1), Romania (1), Serbia (1), Madagascar (2), South Corea (2), Ukraine (2), Spain (3), Yemen (3), Canary Island (4), Vietnam (4), Mexico (9), Turkey (11), USA (11), Hungary (13), Italy (65), SB (40)
*C. annuum* var. *glabriusculum*	SB (2)
C. *baccatum* var. *baccatum*	Perù (2), Bolivia (1), SB (2)
C. *baccatum* var. *pendulum*	Chile (1), Cuba (1), Ethiopia (1), Paraguay (1), Bolivia (4), Brazil (5), Perù (10), SB (10)
*C. chacoense*	Argentina (1), Bolivia (4), SB (2)
*C. chinense*	Bali (1), Bangladesh (1), Burkina Faso (1), Central African Republic (1), Chile (1), Costa Rica (1), Guatemala (1), Vanuatu Islands (1), Barbados Islands (1), USA (1), Yemen (1), Bolivia (2), Maldives (2), Jamaica (2), Mexico (3), Trinidad (3), Caribbean (4), India (5), Perù (7), Brazil (8), SB (10)
*C. eximium*	India (1)
*C. frutescens*	Ecuador (1), Philippines (1), Hungary (1), kenya (1), Perù (1), Portugal (1), USA (1), Brazil (2), SB (3)
*C. pubescens*	Bolivia (1), Cuba (1), Ecuador (1), SB (2), Guatemala (2), Perù (3)

* SB = Seeds Banks.

## Supplementary Materials

The following are available online at http://www.mdpi.com/2223-7747/7/4/103/s1, Figure S1: Distribution of fruit traits in the 307 pepper genotypes under study, Figure S2a: Loading plot of the first and second component based on eight highly correlated fruit traits in all species under study, Figure S2b: Loading plot of the first and second component based on eight highly fruit correlated traits in domesticated and wild species, Figure S3: Hierarchical clustering based on eight highly correlated fruit traits and two most significant plant traits, Table S1: Mean, range, significance of the means within each species and among the 9 Capsicum species for plant traits (Bioversity International), fruit descriptors, Table S2 Variable contribution(VarPC) and correlation coefficient (CorrPC) for fruit descriptors in the two first principal component, Table S3 Variable contribution (VarPC) and Correlation (Corr) for fruit descriptors in the components 3 to 38.
