# Supplementary material for: Large Scale Phenotyping Provides Insight into the Diversity of Vegetative and Reproductive Organs in a Wide Collection of Wild and Domesticated Peppers (Capsicum spp.)
Source: Plants (Basel). 2018 Nov 19;7(4):103. doi: 10.3390/plants7040103 (PMC6313902; doi:10.3390/plants7040103)
Supplement: Supplementary file 1 [file plants-07-00103-s001.zip › plants-382870-supplementary/plants-382870-supp figure.docx]

Article

Large scale Phenotyping Provides Insight into the Diversity of Vegetative and Reproductive Organs in a Wide Collection of Wild and Domesticated Peppers (*Capsicum* spp.)

Pasquale Tripodi * and Barbara Greco


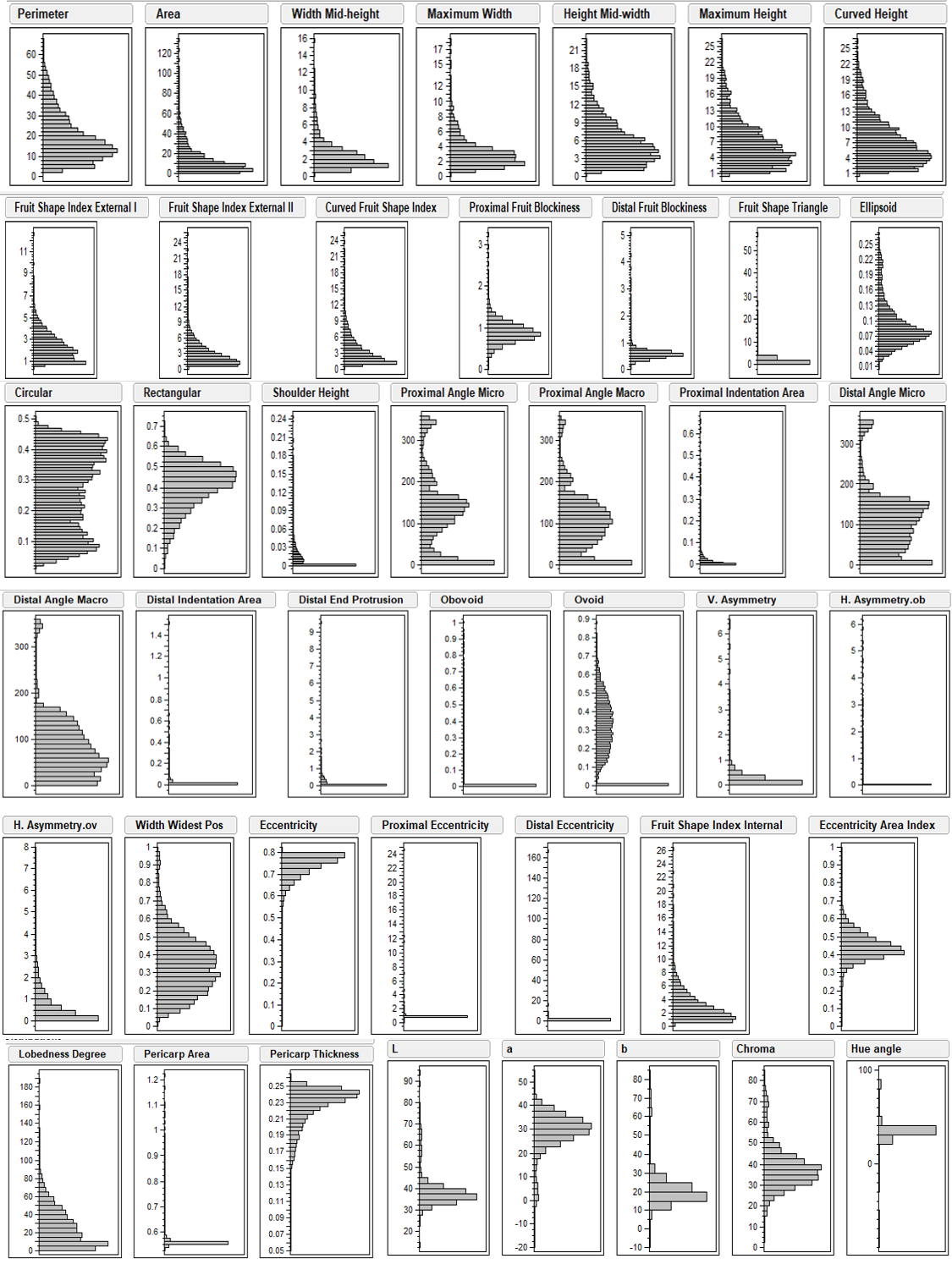


**Figure S1.** Distribution of fruit traits in the 307 pepper genotypes under study.


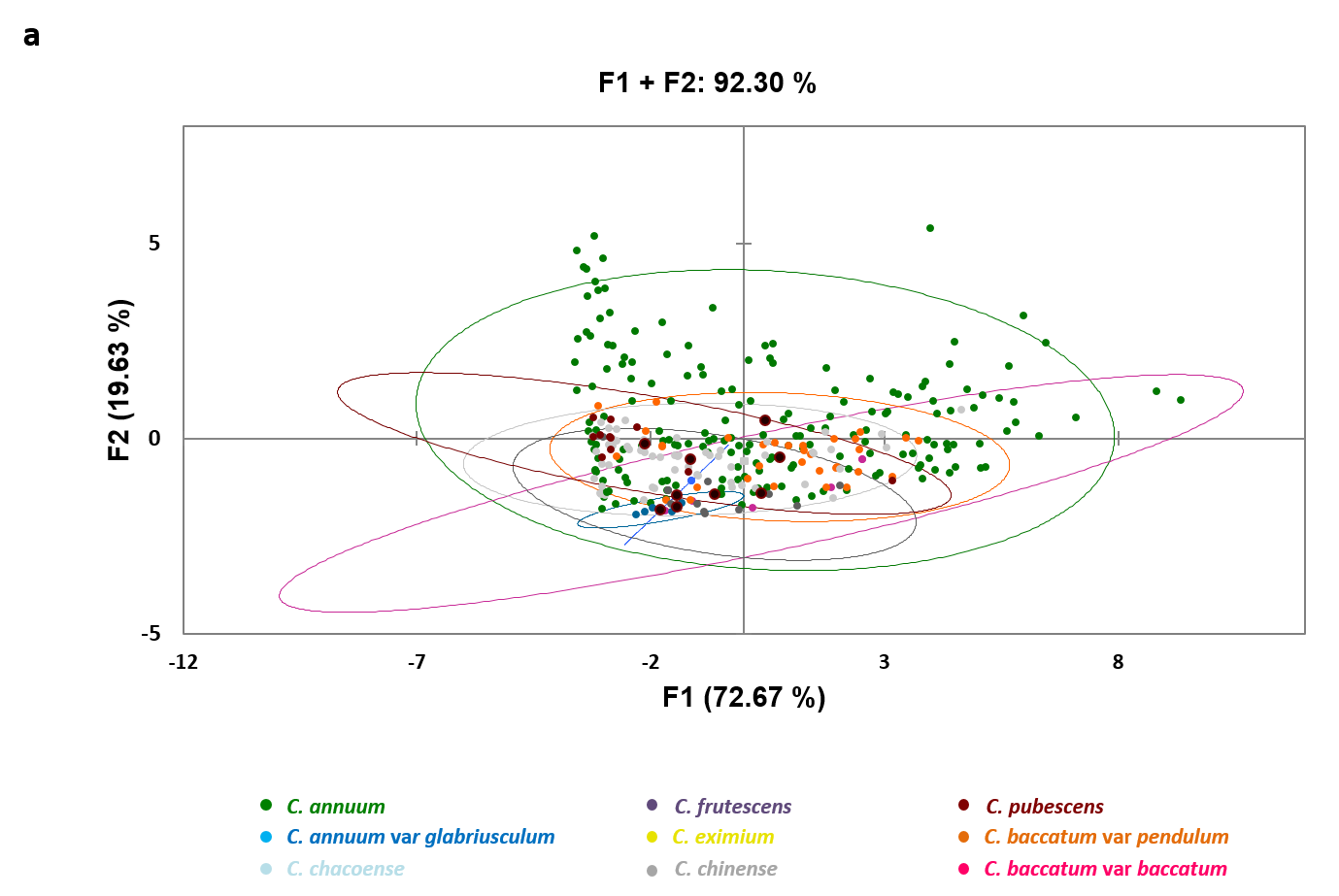

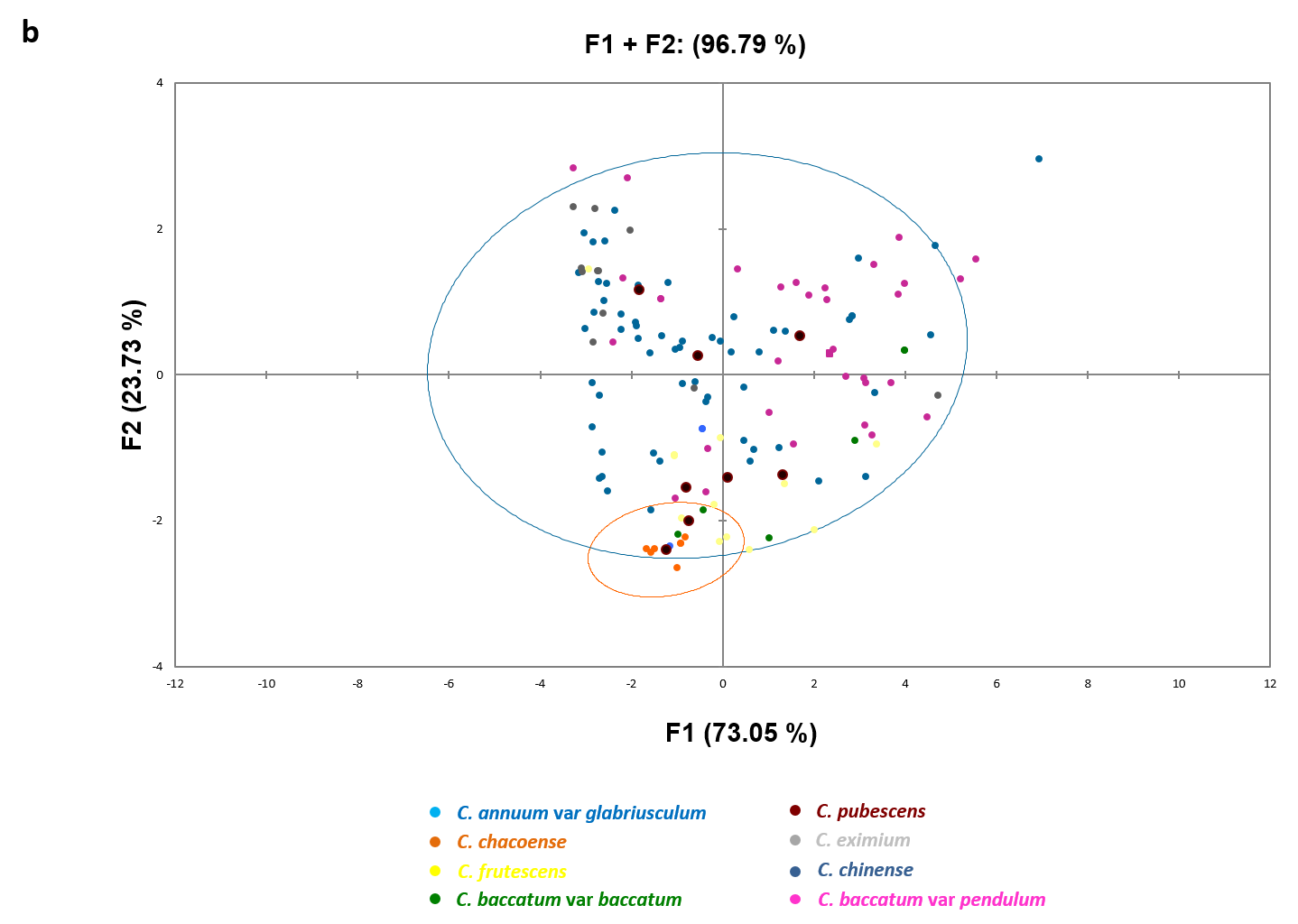


**Figure S2a.** Loading plot of the first and second component based on eight highly correlated fruit traits in all species under study, **Figure S2b**: Loading plot of the first and second component based on eight highly fruit correlated traits in domesticated and wild species.


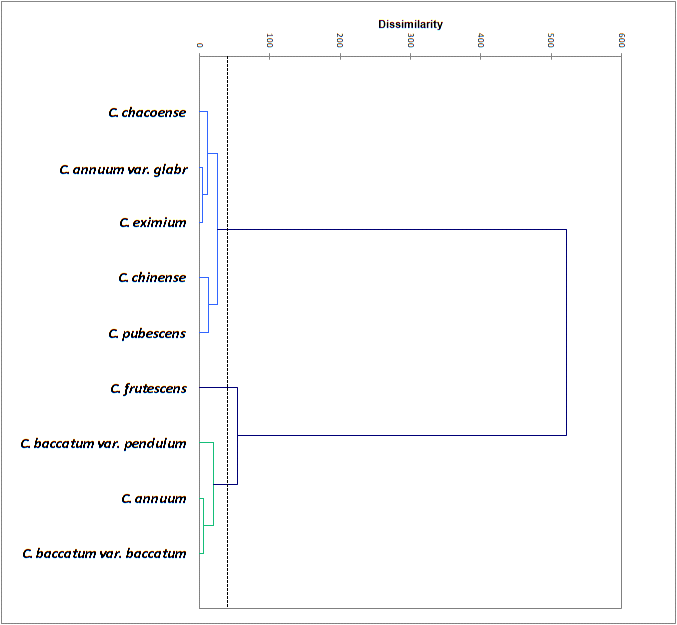


**Figure S3.** Hierarchical clustering based on eight highly correlated fruit traits and two most significant plant traits.
